# Does a history of smoking increase the risk of developing systemic sclerosis? Insights from the Leiden Combined Care in Systemic Sclerosis cohort

**DOI:** 10.1093/rap/rkae055

**Published:** 2024-04-16

**Authors:** Jacopo Ciaffi, Sophie I E Liem, Suzanne C Cannegieter, Saad Ahmed, Eva Hoekstra, Tom Huizinga, Jeska De Vries-Bouwstra

**Affiliations:** Medicine and Rheumatology Unit, IRCCS Istituto Ortopedico Rizzoli, Bologna, Italy; Alma Mater Studiorum – Università di Bologna, DIBINEM, Bologna, Italy; Department of Rheumatology, Leiden University Medical Center, Leiden, The Netherlands; Department of Medicine – Thrombosis and Hemostasis, Leiden University Medical Center, Leiden, The Netherlands; Department of Clinical Epidemiology, Leiden University Medical Center, Leiden, The Netherlands; Department of Rheumatology, Leiden University Medical Center, Leiden, The Netherlands; Department of Rheumatology, Leiden University Medical Center, Leiden, The Netherlands; Department of Rheumatology, Leiden University Medical Center, Leiden, The Netherlands; Department of Rheumatology, Leiden University Medical Center, Leiden, The Netherlands

Key message

- Smoking does not increase the risk of developing systemic sclerosis in the Dutch population.

Dear Editor, Numerous environmental and occupational agents have been linked with the development of SSc [1]. The spectrum of factors potentially acting as triggers in genetically predisposed individuals is broad, including organic solvents, vinyl chloride, pesticides, silicone, epoxy resins, welding fumes and hair dyes [2]. Exposure to silica, in particular, has been suggested to have a fundamental pathogenetic role and is associated with specific clinical peculiarities [3].

With regards to smoking, one study from the USA including 621 patients showed that ever smoking was not associated with the risk of developing SSc [4]. No other study has addressed this issue. Therefore, we decided to investigate whether smoking is a risk factor for the development of SSc in a European population.

We used data from patients followed in the Combined Care in Systemic Sclerosis (CCISS) cohort at Leiden University Medical Center, Leiden, The Netherlands [5]. All patients met the ACR/EULAR 2013 classification criteria for SSc [6]. Smoking status is routinely recorded but information regarding the intensity and duration of tobacco exposure is not always available. Based on smoking status at the time of enrolment, patients were categorized as ‘ever-smokers’ if they were current or former smokers or as ‘never-smokers’. Research on the CCISS cohort is approved by the Ethics Committee of Leiden University Medical Center (CME no. B16.037, REU 043/SH/sh, P09.003/SH/s) and all patients provided written informed consent.

To evaluate the impact of smoking on the risk of developing SSc, the smoking status of SSc patients from the Leiden CCISS cohort was compared, using a chi-squared test, with the prevalence of smoking in the overall Dutch population in two different years, 2015 and 2021. Data regarding the percentage of ever-smokers and never-smokers and the population size in the Netherlands in the same years were gathered from the Dutch Central Bureau of Statistics (CBS) [7, 8]. Since CBS data are stratified by sex and age range (25–44 years, 45–64 years, ≥65 years), CCISS data were categorized accordingly.

All patients from the CCISS cohort ≥25 years of age who were alive and in follow-up in the two reference years were included. In 2015, there were 277 patients in follow-up in the CCISS cohort with an age ≥25 years. Of these, 229 (83%) were females and 147 (53%) were ever-smokers. In 2021, there were 364 patients in follow-up with an age ≥25 years, but 2 were excluded because information about smoking was missing. Of the 362 included patients, 284 (79%) were females and 184 (51%) were ever-smokers.

The prevalence of ever-smokers in the CCISS cohort and in the general Dutch population in 2015 and 2021 is shown in Table 1. In 2015, the proportion of ever-smokers among male patients was 82% in the 25–44 years group, 56% in the 45–64 years group and 67% in the ≥65 years group. Among female patients, this was respectively 43%, 55% and 50%. The prevalence of ever-smoking SSc patients was comparable to the prevalence of ever-smokers in the Dutch population (Table 1).

**Table 1.** Prevalence of ever smoking in men and women in the Dutch population and in SSc patients followed in the Leiden CCISS cohort in 2015 and 2021

Age range (years)	Dutch population (ever-smokers), *n* (%)	SSc patients (ever-smokers), *n* (%)	*P*-value
2015			
Men			
25–44	2 126 000 (55)	11 (82)	0.078
45–64	2 385 000 (65)	25 (56)	0.345
≥65	1 362 000 (79)	12 (67)	0.291
Women			
25–44	2 116 000 (48)	49 (43)	0.492
45–64	2 369 000 (61)	106 (55)	0.198
≥65	1 644 000 (52)	74 (50)	0.705
2021			
Men			
25–44	2 193 000 (52)	9 (56)	0.820
45–64	2 409 000 (60)	53 (70)	0.127
≥65	1 604 000 (77)	16 (56)	0.054
Women			
25–44	2 154 000 (43)	41 (34)	0.240
45–64	2 413 000 (54)	152 (45)	0.043
≥65	1 849 000 (55)	91 (55)	0.985

Weighting the age categories, in 2015 the overall prevalence of ever smoking in men was 65% in both the general population and in SSc patients (*P* = 0.995), while in women it was 54% and 51%, respectively (*P* = 0.296).

In 2021, the prevalence of ever-smokers among male patients was 56% in the 25–44 years group, 70% in the 45–64 years group and 56% in the ≥65 years group. Among female patients, this was respectively 34%, 45% and 55%. In female SSc patients ages 45–64 years, the proportion of ever-smokers was lower than in the Dutch population (*P* = 0.043). No other statistically significant differences emerged (Table 1).

Weighting the age categories, in 2021 the overall prevalence of ever smoking in men was 62% in the general population and 66% in SSc patients (*P* = 0.488), while in women it was 51% and 47%, respectively (*P* = 0.165).

Our analysis comparing the prevalence of smoking in SSc patients with that in the general population revealed no significant differences. The proportion of individuals who have ever smoked is similar among both patients with the disease and the control group. Thus we can confirm that, in line with the findings of Chaudhary *et al.* [4] in the USA, smoking does not appear to increase the risk of developing SSc in the Dutch population.

Some limitations need to be acknowledged. We lacked data on smoking intensity and duration for all patients, preventing us from assessing dose–response effects and comparing smoking duration or abstinence between SSc patients and the general population. Additionally, the self-reported nature of smoking data is prone to recall bias. Smoking information was collected at baseline, yet the impact of smoking on disease onset may change over time, with the risk potentially influenced by both the extent and duration of smoking habits.

In conclusion, the prevalence of ever-smokers among Dutch SSc patients aligns with that of the general population in the Netherlands. Our findings do not point towards a role of smoking exposure in the development of SSc.

## Data Availability

The data that support the findings of this study are available upon reasonable request.
